# Chemical Characterization and Antibacterial Properties of *Fontitrygon margarita* (Günther, 1870) Liver Oil

**DOI:** 10.1155/2022/9369387

**Published:** 2022-07-30

**Authors:** Boris Simo Noutsa, Sammuel Raymond Tchabong, Arlette Danelle Deutchoua Djitieu, Fabrice Fabien Dongho Dongmo, Fabrice Hervé Njike Ngamga, Ronice Zokou, Ousman Tamgue, Rosalie Anne Ngono Ngane, François Tchoumbougnang

**Affiliations:** ^1^Department of Processing and Quality Control of Aquatic Products, Institute of Fisheries and Aquatic Sciences, University of Douala, PO Box 7236, Douala, Cameroon; ^2^Laboratory of Analytical Chemistry, Faculty of Sciences, University of Yaoundé I, B. P. 812 Yaoundé, Cameroon; ^3^Research Unit of Biochemistry of Medicinal Plants, Food Sciences and Nutrition, University of Dschang, P.O. Box 67, Dschang, Cameroon; ^4^Department of Biochemistry, Faculty of Science, University of Douala, P.O. Box 24157 Douala, Cameroon

## Abstract

**Objective:**

The aim of this study was to determine the chemical characteristics and antibacterial activity of *Fontitrygon margarita* liver oil against the bacteria responsible for food poisoning.

**Methods:**

Oils were extracted from *F. margarita* liver using two methods (exudation and cooking-pressing) and analyses by Fourier transform infrared (FTIR) spectroscopy. Quality indexes were determined using standard methods and the fatty acid profile was carried out by gas chromatography with a flame ionization detector (GC-FID). Antibacterial activities of these oils, their emulsion, and their interactions with common antibiotics were evaluated by the broth microdilution method.

**Results:**

Extraction yield was higher with cooking-pressing (16.90%) compared to exudation (14.49%). The quality indexes of both oils were conformed to Codex Alimentarius Standard. Thiobarbituric acid index was higher with exudation compared to cooking-pressing (3.20 ± 0.14 and 2.36 ± 0.14 *μ*mol MDA/kg, respectively) while acid, iodine, peroxide, and anisidine values did not significantly vary with the extraction methods (2.15-2.30 mgKOH/g, 102.42-106.65 gI_2_/100 g, 3.34-3.57 meqO_2_/kg, and 2.85-3.32 respectively). FTIR analyses clearly show that the two spectra are similar (no differences in the frequency and absorbance of their bands). The fatty acid profile revealed that, regardless of the extraction methods, *F. margarita* oil is richer in monounsaturated (55.97-55.41%) followed by polyunsaturated (28.17-28.52%) and saturated fatty acids (15.86-16.07%). Moreover, these oils showed antibacterial activity on all the bacteria strains tested with MICs between 16 and 256 mg/ml. Regardless of the extraction methods, emulsions showed higher activity (6.25 ≤ MIC ≤25 mg/ml) compared to crude oils. Additionally, *F. margarita* liver oil potentiated the antibacterial activity of ciprofloxacin, tetracycline, gentamicin, amoxicillin, and chloramphenicol.

**Conclusion:**

These results showed the effectiveness of *Fontitrygon margarita* liver oil against some bacteria responsible for food poisoning, thus demonstrating their antibacterial properties which could be due to their chemical composition.

## 1. Introduction

Food provides energy and substances necessary for body functioning. They are likely to be contaminated. These contaminated foods, once ingested by humans, can cause food poisoning which is mainly due to bacterial germs such as *Staphylococcus aureus*, *Salmonella sp.*, *Escherichia Coli*, and *Clostridium perfrigens* [1, 2]. In 2015, the global burden of foodborne disease indicates that each year up to 600 million people or one in ten people becomes ill after consuming contaminated food, of whom 410,000 die, including 125,000 children under five (30% mortality) [1]. The most effective drug weapons used against these bacterial infections are antibiotics. However, the abusive and inappropriate use of these pharmaceutical products causes problems of biodegradability in the body and bioresistance of bacteria, which thus constitutes a brake on the control of bacterial infections and the origin of therapeutic failures. In the search for alternatives, some researchers have opted for a possible use of fish oils as antibacterial. In fact, fish oil is the richest source of long-chain polyunsaturated fatty acids including Eicosapentaenoic acid (EPA, C20: 5n-3) and Docosahexaenoic acid (DHA, 22: 6n-3). Indeed, antibacterial activity of fatty acids is accepted nowadays and their prime target being the cell membrane, where they disrupt the electron transport chain and oxidative phosphorylation. Besides interfering with cellular energy production, fatty acid action may also result from the inhibition of enzyme activity, impairment of nutrient uptake, generation of peroxidation, and auto-oxidation degradation products or direct lysis of bacterial cells [3]. Long-chain unsaturated fatty acids have greater potency compared to short and medium chain or saturated fatty acids. Besides, Mil-Homens et al. [3] showed that docohexaenoic *ω*3 fatty acid had antibacterial property. Likewise, Chitra and Radhakrishnan [4] showed that polyunsatured fatty acid extracts from *Sardinella longiceps* and *Sardinella fimbriata* had antibacterial activities. Also, our lasted researches showed that oils from *Lutjanus dentatus* [2], *Hepsetus odoe* [5], and *Chrysichthys nigrodigitatus* [5, 6] had antibacterial properties.Global fish production from fisheries and aquaculture is estimated to 171 million tons [2, 7]. A large part of this production (151 million tons) is processed for human consumption [7]. This processing generates a significant amount of offal and other by-products among which viscera, bones, fins, skins, fat, and livers can represent up to 70% of the volume of fish used in industrial processing [8]. These co-products are still under-exploited while they are a source of nutrients and compounds with therapeutic and functional properties which will be useful for humans [9]. In Cameroon, these co-products are used for animals fed or are simply released into the environment.At the pretreatment unit of Youpwe landing stage in Douala city, many species of fish are encountered including *Fontitrygon margarita*, from which 1,156 kg are cleaned each month between 2013 and 2018, generating an average of 28 kg of *F. margarita* livers in the same period and their co-products among which livers are directly discharged into the sides of the coast of the Wouri River, causing pollution problems. Faced with this risk of environmental pollution and the growing demand of fish, special attention should be paid to by-products including livers which are potential sources of polyunsaturated fatty acids [10]. In fact, fish liver oil is the richest source of long-chain polyunsaturated fatty acids, including EPA and DHA. They could be an alternative of dietary supplement against bacteria responsible for food poisoning diseases. Given the insolubility of lipids in water, the antibacterial activity of oil could be improved through the production of emulsions. Indeed, many studies showed the effectiveness of nanoemulsions to increase the bioavailability of hydrophobic active substances [11]. Furthermore, oil could be obtained by different extraction methods among which are enzymatic extraction, chemical extraction, and physical extraction. It is showed that extraction yield and quality of fish oil depend on extraction method [2, 5, 6].

In order to promote the use of natural product for fighting against bacteria responsible of food poisoning and to contribute to the protection of environment, the objective of this study is to determine the chemical characteristics of *Fontitrygon margarita* liver oil extract with exudation and cooking-pressing, and the antibacterial activity of this oil and its nanoemulsion.

## 2. Materials and Methods

### 2.1. Materials

#### 2.1.1. Collection of Livers of *Fontitrygon margarita*

*Fontitrygon margarita* livers were collected from fish cleaners at Youpwe landing stage in Douala during April 2020. Indeed, livers collected each day were kept at -4°C. At the end of collection period, about 5 Kg of livers were obtained. They were taken under ice to the Valorization and Quality Control Laboratory of the Institute of Fisheries and Aquatic Sciences of the University of Douala in Yabassi where extraction was done immediately.

#### 2.1.2. Microorganisms

The bacteria used in this study consisted of Gram^+^ and Gram^−^ bacteria, including strains of *Staphylococcus aureus*, *Escherichia serovar typhi*, *Citrobacter freundii*, and *Yersinia enterocolitica*. The reference strains (ATCC 8739, ATCC 10536, ATCC 27853, and ATCC 28579) were obtained from the Laboratory of Microbiology and Antimicrobial Substances of the University of Dschang; meanwhile, the clinical strains (EC 6, EC 137, ENT 119, ENT 163, ENT 51, PSNEA, KLPB 101, KL 11, SAL 9, CITB 80, CITB 81, YERB 121, YERB 1, SERB 115, ST 120, and MRSA 3) were obtained from the Laboratory of Biochemistry of the University of Douala.

### 2.2. Methods

#### 2.2.1. Oils Extraction

Two methods were used to extract oil, namely, exudation and cooking-pressing methods. For this purpose, livers were divided into two equal parts, one part for each extraction method.

*(1) Exudation Method*. The livers of *Fontitrygon margarita*, cut into pieces, were placed in an oven for 10 min at a temperature of 95°C [12]. After drying, oil was weighed and stored in dark vials at 4°C after adding anhydrous sodium sulfate to remove all traces of moisture.

*(2) Cooking-Pressing Method*. Livers were cooked at 95°C in a household autoclave containing water on a Kinderbo gas-type plate for 10 min. The resulting pressed juice was decanted for 45 min and centrifuged 15 min at 2490 g (TT-4500 low speed centrifuge, Hercuvan Lab Systems). Anhydrous sodium sulfate was added to remove all traces of moisture; then, oils were weighted, kept in dark vials, and stored at 4°C until use [2, 12].

#### 2.2.2. Determination of Fish Oil Chemical Quality

*(1) Fish Oil Quality Indexes*. Acid, iodine, and anisidine values were evaluated according to standard methods recommended by the French Association for Standardization [13]. Peroxide value was determined by the spectrophotometry method recommended by the International Dairy Federation [14]. Thiobarbituric acid index was determined using the method recommended by the American Oil Chemist Society [15] and total oxidation by calculation using the formula:

TOTOX = 2 Peroxide value + Anisidine value.

All the analyses were done in triplicate.

*(2) Fourier Transform Infrared (FTIR) Spectra Analyses*. IR spectra between 3800 and 500 cm^−1^ were recorded using a tensor 27 (Bruker, Wissemberg, France) equipped with an ATR prism crystal accessory and MCT detector (Mercury Cadmium Telluride). The spectra resolution was 4 cm^−1^. Measurements were performed at RT using approximately 2 *μ*l of the fish oils, which were placed on the surface of the ATR crystal and pressed with a flat-tip plunger until spectra with suitable peaks were obtained. The background was subtracted using the spectrum software OPUS version 6.3.2 (Perkin-Elmer Inc.).

*(3) Fatty Acid Composition*. Fatty acid composition of oil samples was determined by gas chromatography coupled to a flame ionization detector (GC-FID). Briefly, the fatty acid methyl esters were prepared by trans-esterification, using 2% sulfuric acid in methanol [16]. Analyses were performed on a gas chromatograph (Agilent Technologies, Palo Alto, CA, USA), serial number 7890A, coupled to a flame ionization detector, using a capillary column DB-225 (30 m ×0.25 mm, film thickness 0.25 *μ*m). The initial column temperature was 160°C for 2 min, then increased to 220°C (5°C/min) and maintained for 10 min. Nitrogen was used as mobile phase with flow rate 1.5 ml/min. The temperature of the injector and detector were maintained at 230°C and 250°C, respectively, for 20 min. Fatty acids were identified by peak retention time and elution order and compared against a reference fatty acid methyl esters standard test mix (Fatty Acid Methyl Esters Standard Mixture SMB00937) and a marine test mix PUFA No.1 (Marine Source, Analytical Standards), both from Sigma-Aldrich, Castle Hill, Australia.

#### 2.2.3. Antibacterial Activity

Antibacterial activity consisted of determining the activity of crude fish oils and their nanoemulsions, and the interaction of oils and emulsions with common antibiotics.

*(1) Preparation of Stock Solutions of Oils, Nanoemulsion, and Antibiotics*. Stock solutions of oils were prepared at 1024 mg/ml in a solution of Tween 80 (5%). Stock solution of oil nanoemulsions was prepared at 50 mg/ml using Tween 80 as emulsifier according to the protocol described by Prinderre et al. [17]. Antibiotics (Ciprofloxacin, Chloramphenicol, Amoxicillin, Tetracycline, and Gentamicin) were prepared at a concentration of 256 *μ*g/ml.

*(2) Antibacterial Activity Evaluation of Fish Oils and Nanoemulsion*. The antibacterial activity of fish oils and their nanoemulsion was evaluated by the broth microdilution method in 96-well microplates [18]. For this purpose, 100 *μ*l of Mueller-Hinton broth was added in each well of plates. Then, 100 *μ*l of oil or nanoemulsion was introduced in the first well of each plate. Series of twofold dilutions were performed to obtain a final concentration range from 2 to 256 mg/ml in a total volume of 100 *μ*l/well. In parallel, bacterial suspensions of about 1.5 × 10^8^ CFU/ml (Mc Farland turbidity standard no. 0.5) were prepared from an overnight culture. The inoculum was obtained following 100 times dilution (1.5 × 10^6^ CFU/ml). A volume of 100 *μ*l of inoculum was added in each well of previous plates which were further incubated at 35°C for 18 hours. Growth was monitored using p-iodo tetrazolium chloride (INT; 0.2 mg/ml) [19]. Viable bacteria change the yellow dye of iodonitrotetrazolium into a pink color. The lowest concentration of oil, at which no visible color change was noted, was considered Minimum Inhibitory Concentration (MIC). Ciprofloxacin was used as positive control.

Minimum Bactericidal Concentration (MBC) was determined by adding 50 *μ*l aliquots of the preparations which did not show any growth after incubation during MIC assays to 150 *μ*l broth culture medium. These preparations were incubated at 37°C for 24 hours. The MBCs values were defined as the lowest concentration of test samples that prevent from color change of INT as mentioned above. Following MBCs determinations, the MBC/MIC ratio is allowed to reveal either the bactericidal effect (MBC/MIC ≤4) or the bacteriostatic effect (MBC/MIC>4) [20].

*(3) Fish Oils–Antibiotics Interaction Study*. 100 *μ*l of Mueller Hinton broth culture medium was introduced into each well of a 96-well microplate, followed by the addition of 100 *μ*l of antibiotic. A subsequent dilution was thereafter made to obtain final antibiotic concentrations less than or equal to the MIC. 100 *μ*l of oil solution was introduced into each well. The oil stock solutions were prepared so as to obtain final concentrations in the wells equal to MIC and MIC ×2. The content of each well was diluted by adding 100 *μ*l of inoculum. The MIC was determined as mentioned above [19]. The interaction between each oil sample and the antibiotics including Ciprofloxacin, Chloramphenicol, Amoxicillin, Tetracycline, and Gentamicin was determined by calculating the Fractional Inhibitory Concentrations (FIC) using the formula [21]:

FIC = MIC_ATB+Oil_/MIC_ATB alone_.

Four types of interactions were recorded according to the value of the Fractional Inhibitory Concentration: synergistic interaction (FIC ≤0.5); additive interaction (0.5 < FIC ≤1); indifferent interaction (1 < FIC ≤4); and antagonistic interaction (FIC>4).

### 2.3. Statistical Analyses

The results were expressed as mean ± standard deviation. The means were compared by the ANOVA *I* test to the 5% probability threshold using GraphPad InStat software version 2000.

## 3. Results

### 3.1. Extraction Yield

As shown in Table 1, the extraction method of liver oil significantly affected the yield of oil. The highest yield (16.9%) was obtained by cooking-pressing method.

### 3.2. Chemical Quality of *Fontitrygon margarita* Liver Oil

#### 3.2.1. Quality Indexes

Acid, iodine, peroxide, anisidine, and total oxidation values of the oil extracted according to the two extraction methods used are not significantly different at the threshold of 5% (Table 1). However, these oil samples remain compliant with the Alimentarius Codex 2017 Standard.

#### 3.2.2. Fatty Acid Profile

Table 2 shows that *Fontitrygon margarita* liver oil contains 26 fatty acids regardless of extraction methods including saturated, monounsaturated, and polyunsaturated fatty acids. The liver oil of *F. margarita* is richer in monounsaturated fatty acids (55.97 and 55.41%), polyunsaturated fatty acids (28.17 and 28.52%), and saturated fatty acids (15.86 and 16.07%), respectively, for extraction by cooking-pressing and exudation.

In saturated fatty acids, palmitic acid is by far the most concentrated with contents varying between 9.96 and 9.94%, followed by myristic acid 2.73 and 2.64%. In monounsaturated fatty acids, oleic acid is the most abundant with contents ranging between 41.9% and 41.23%, followed by eicosatetraenoic acid between 4.45% and 4.42%. Among the polyunsaturated fatty acids, linoleic acid (LA) (*ω*6), *α*-linolenic acid (*ω*3), docosahexaenoic acid (DHA) (*ω*3), and eicosapentaenoic acid (EPA) (*ω*3) are the most predominant. However, the gamma linolenic acid and heptadecenoïc acid content are twice as high in oil obtained by exudation (0.32% and 0.34%, respectively) compared to oil extracted by cooking-pressing (0.15% and 0.17%, respectively). In addition, lauric acid is four times higher in oil obtained by exudation (0.12% against 0.03%). The sum of omega-6 is slightly higher in oil obtained by exudation 14.34% compared to oil extracted by cooking-pressing 13.87%. The ratio PUFA/AGS is 1.77 for this fish oil.

#### 3.2.3. FTIR Spectra of Oils

Infrared spectroscopy was used to identify the functional groups present in the organic components of samples of *Fontitrygon margarita* liver oils. From a global point of view, these spectra have the same shape, as originated from the same fish.

**Figure 1:**
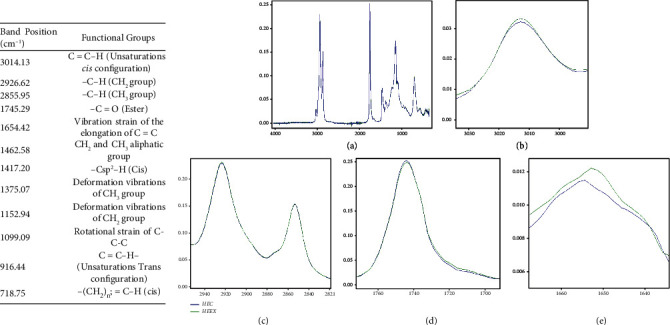
FTIR profile of *Fontitrygon margarita* liver oils according to the extraction method. (a) General shape of the curves between 4000 and 500 cm^−1^. (b) Region curves between 3040 and 2900 cm^−1^. (c) Region curves between 2953 and 2820 cm^−1^. (d) Region curves between 1772 and 1774 cm^−1^. (e) Region curves between 1667 and 1661 cm^−1^; HEC: oil extracted by cooking-pressing; HEEX: oil extracted by exudation.

The spectra obtained between 3800 and 500 cm^−1^ on oils extracted by exudation and cooking-pressing at 95°C are presented in Figure 1(a). It is observed that the two spectra are similar. This means that the nature and content of the chemical compounds present in the oils are not significantly different. This correlated with the fatty acid composition result.

The absence of a band between 3800 cm^−1^ and 3100 cm^−1^ on the spectra of different oils indicates the absence of hydroxyl groups in the compounds contained in oils.

The peak at 3014 cm^−1^, linked to the Csp^2^-H stretching vibration of the cis double bond (= CH), provides information on the degree of lipid unsaturation. The two peaks just below 3000 (2926 cm^−1^ and 2855 cm^−1^) can both be attributed to the absorption caused by the asymmetrical and symmetrical stretching vibrations of the methyl and methylene groups (Figure 1(c)) which characterize the long carbon chains of fatty acids. The stretch vibration band assignable to the C=O ester group of triglycerides was found around 1745 cm^−1^ (Figure 1(d)). The weak peak at 1654 cm^−1^ (Figure 1(e)) attributed to the vibration strain of the elongation of C=C (cis), also reflects the double bond present in unsaturated fatty acid oil. The bands observed in fingerprint regions below 1500 cm^−1^ allow accurately characterizing the molecules. Therefore, the band at 1462 cm^−1^ is assigned to the bending vibrations of the CH_2_ and CH_3_ aliphatic groups. The peak appears near 1417 cm^−1^ attributed to rocking vibrations of Csp^2^-H bonds of Cis-disubstituted olefins. The bands at 1375 cm^−1^ and 1153 cm^−1^ are due, respectively, to symmetrical bending vibration of CH_3_ and CH_2_ groups. The bands appearing at 916 cm^−1^ (Figure 1(d)) are linked to the vibration in the molecules analyzed of the double trans bonds, while those appearing at 718 cm^−1^ (Figure 1(d)) are linked to the vibration in the molecules analyzed of the double cis bonds.

### 3.3. Antibacterial Properties of *Fontitrygon margarita* Liver Oil and Interactions with Some Antibiotics

#### 3.3.1. Antibacterial Properties of Oil and Nanoemulsion

The antibacterial activity of the oils varied depending on the extraction method, bacterial strains, and the preparation technique of the test sample (Table 3). The two oils showed comparable antibacterial activity against strains of *E. Coli* and *E. Cloacae* 16 ≤ MIC ≤64 mg/ml and in particular on strains resistant to methicillin MIC =128 mg/ml. With MIC values of 32 mg/ml on *P. aeruginosa* strains, 16 ≤ MIC≤32 mg/ml on K*. Pneumoniae* and *Y*. *enterocolitica*, 16 ≤ MIC≤64 mg/ml on *Citrobacter freundii*, and confirmed activity on all the strains of *Salmonella enterica serovar typhi*, the oil obtained by exudation was more active than that obtained by cooking-pressing. In general, the MICs value of the nanoemulsions was lower regardless of the oil extraction method (6.25 ≤ MICs ≤25), reflecting an increase of antibacterial activity. The activity of these emulsions increases by 10 times on most bacteria.

In general, the MICs value of the emulsions was lower regardless of the oil extraction method (6.25 ≤ MIC ≤25), reflecting an increase of antibacterial activity. The activity of these emulsions increases by 10 times on most bacteria.

Regardless of the bacteria strain and the technique of preparation of the stock solution to be tested, the MBC/MIC ratio was greater than or equal to 4 in general, reflecting a bacteriostatic activity of liver oil from *Fontitrygon margarita*.

#### 3.3.2. Interactions with Some Antibiotics

The combination of oils with antibiotics showed that liver oil from *Fontitrygon margarita* potentiated the activity of Ciprofloxacin on strains of *K. pneumoniae*, *S. typhi*, and *S .aureus*, Amoxicillin on *E. cloacae* and *Y. enterocolitica*, Tetracycline on *E. coli*, *K. pneumoniae*, *S. typhi*, and *C. freundii*, Gentamicin on *K. pneumoniae*, *C. freundii*, and *S .aureus*, and Chloramphenicol on *K. pneumoniae*, *C .freundii*, and *Y. enterocolitica*. On the other hand, the antagonistic and indifferent effects were observed with few bacterial strains (Table 4).

## 4. Discussion

The cooking-pressing method made it possible to obtain the liver oil from *F. margarita* with a better yield than with the exudation method. This increase could be attributed to the high pressurized steam which could favor the release of the oil from the tissues [2, 12]. The acid number provides information on the amount of free fatty acids present in oil. The acid number of *F. margarita* liver oil extracted by both methods was compliant with the standard (≤3 mg KOH/g oil) [22]. The low content of free fatty acids obtained could be due to the deactivation of lipolytic enzymes by heat, thus preventing the hydrolysis of triglycerides in the oil. This justifies the absence of the characteristic peak of the hydroxyl group (OH) in the range 3800–3100 cm^−1^. These results are in agreement with those obtained by Weber et al., [23]. According to the last, the cooking-pressing methods applied to fish resulted in a significant reduction in its content of free fatty acids.

The iodine value provides the information on the number of fatty acid double bonds [24]. The oil extracted by cooking-pressing had an iodine value slightly lower although not significant (*P* > 0.05) than that obtained by exudation. Likewise, they peak at 3014 cm^−1^, linked to the CH stretching vibration of the cis double bond (= CH), which provides information on the degree of lipid unsaturation have the same intensity (Figure 1(b)). A similar peak, related to the C-H stretching vibration of the cis double bond (= CH), was obtained in salmon lipids and catfish oils at 3010 cm^−1^ [25, 26] and mackerel fish lipid at 3012 cm^−1^ [27].

The values of the peroxide index of the skate liver oil extracted according to both methods are not significantly different (*P* > 0.05) and remain in conformity with the Codex Alimentarius Standard (5 meqO_2_/kg). This could be due to a limited exposure of the oil to heat (10 min) thus limiting the reaction of oxygen with double bonds.

The thiobarbituric acid index of *F. margarita* liver oil obtained by exudation is higher than those from oil obtained by cooking-pressing. However, according to food standards, the oils were of good quality because they had indexes below 10 *μ*mol MDA/kg.

The evaluation of the antibacterial activity of liver oils of *F. margarita* obtained by the two extraction methods revealed MICs values between 16 and 256 mg/ml, reflecting the activities of these oils on bacteria responsible for foodborne diseases. This antibacterial activity could be justified by the presence in these oils of saturated fatty acids and polyunsaturated fatty acids of the family of *ω*3 (linoleic acid, eicosapentaenoic acid, and docosahexaenoic acid) and *ω*6 (linolenic acid and arachidonic acid) [4]. These oils could therefore be used to relieve stomach aches due to contamination by these enterobacteria. These results are in agreement with those obtained by Mouokeu et al. [5] who showed activity of oil from *Chrysichthys nigrodigitatus* (32 ≤ CMI ≤64 mg/ml) and *Hepsetus odoe* (8 ≤ CMI ≤64 mg/ml) on some bacteria responsible for food poisoning.

The oil obtained following exudation was active on a wide range of bacteria compared to that obtained by cooking-pressing. This could be due to the products of lipids peroxidation which, according to MDA content, is significantly higher in oil extracted by exudation than that obtained by cooking-pressing. Indeed, Kuan *et al.* [28] showed the production of reactive oxygen species (ROS) over time, which subsequently caused a significant increase in MDA production (a marker for lipid peroxidation) and ultimately led to cell death by disrupting the integrity of the bacterial membrane.

The antibacterial activity of the nanoemulsions from *F. margarita* liver oil was greater compared to oils used for their preparation. This can be explained by the fact that the emulsions improve the penetration of the active fatty acids through the bacterial cell membrane thanks to their large specific surface and to the reduction of the interfacial tension of the droplets [29]. Indeed, in comparison with microemulsions, nanoemulsions in question in this work have better solubility and much greater specific surface, which gives an optimized diffusibility of the active substance.

Oils rich in polyunsaturated fatty acids behave as adjuvants that can modulate the activity of some antibiotics [30]. The *F. margarita* liver oils potentiated the activity of Ciprofloxacin, Amoxicillin, Tetracycline, Gentamicin, and Chloramphenicol on the bacteria tested with a better potentiation on the strains of *Klebsiella pneumoniae*. The potentiating activity could be explained by the presence of PUFAs including omega 3 and omega 6, saturated fatty acids (myristic and palmitic), and oxidation products of fatty acids which could improve the activity of antibiotics.

## 5. Conclusion

This study showed that the extraction yields as well as the quality indexes do not vary significantly with the extraction method used. The extraction by exudation and by cooking-pressing did not affect the quality of this oil; the quality indexes remained in conformity with Codex Alimentarius Standard. Monounsaturated and polyunsaturated fatty acids are most abundant in *Fontitrygon margarita* liver oil which generally exhibits antibacterial activity. However, the oil extracted by exudation was more active on a wide range of bacteria. Nanoemulsions presented better antibacterial activity compared to those of the oils used for their formulation. *F. margarita* liver oil potentiates the activity of Ciprofloxacin, Amoxicillin, Tetracycline, Gentamicin, and Chloramphenicol on some bacteria responsible for food poisoning.

Antibacterial activity of *Fontitrygon margarita* liver oil is of particular interest to the nutraceutical industry. Thus, this oil could be used as a dietary supplement to improve the effectiveness of some drugs against foodborne infections.

## Figures and Tables

**Table 1:** Extraction yield and quality indexes of *Fontitrygon margarita* liver oil according to the extraction method.

	Extraction yield (%w/w)	Acid value (mgKOH/g)	Iodine value (gI2/100 g)	Thiobarbituric acid value (*μ*mol MDA/kg)	Peroxide value (meqO_2_/kg)	Anisidine value	Totox
Oil extracted by cooking-pressing	14.49 ± 0.80^a^	2.3 ± 0.23^a^	102.42 ± 1.20^a^	2.36 ± 0.14^a^	3.34 ± 0.17^a^	2.85 ± 0.17^a^	9.04 ± 0.27^a^
Oil extracted by exudation	16.90 ± 1.51^b^	2.15 ± 0.84^a^	106.65 ± 5.01^a^	3.2 ± 0.14^b^	3.57 ± 0.34^a^	3.32 ± 0.8^a^	10.21 ± 1.94^a^
Codex Alimentarius Norm 2017	/	≤3		≤10	≤5	≤20	/

In the same column, the numbers with the same letters are not significantly different (*P* > 0.05), *n* =3.

**Table 2:** Fatty acid profile of *Fontitrygon margarita* liver oils according to the extraction method.

Fatty acids	Common name of fatty acid	Oil extracted by cooking-pressing (%)	Oil extracted by exudation (%)
C12:0	Lauric acid	0.03 ± 0.01^a^	0.12 ± 0.05^b^
C14:0	Myristic acid	2.73 ± 0.26^a^	2.64 ± 0.34^a^
C16:0	Palmitic acid	9.96 ± 1.30^a^	9.94 ± 1.96^a^
C17:0	Margaric acid	0.20 ± 0.04^a^	0.25 ± 0.04^a^
C18:0	Stearic acid	2.40 ± 0.24^a^	2.50 ± 0.33^a^
C20:0	Arachidic acid	0.30 ± 0.09^a^	0.40 ± 0.06^a^
C22:0	Béhénic acid	0.16 ± 0.03^a^	0.15 ± 0.04^a^
C24:0	Lignoceric acid	0.08 ± 0.01^a^	0.07 ± 0.01^a^
*ΣSFA*		**15.86 ± 1.57** ^a^	**16.07 ± 2.41** ^a^
C12:1	Lauroleic acid	2.60 ± 0.39^a^	2.58 ± 0.26^a^
C16:1	Palmitoleic acid	3.25 ± 0.64^a^	2.96 ± 0.44^a^
C17:1	Heptadecenoïc acid	0.17 ± 0.05^a^	0.34 ± 0.05^b^
C18:1	Oleic acid	41.90 ± 4.59^a^	41.23 ± 5.19^a^
C20:1 *ω*6	Eicosatetraenoïc acid	4.45 ± 0.44^a^	4.42 ± 1.24^a^
C22:1	Erucic acid	3.25 ± 0.55^a^	3.50 ± 0.69^a^
C24:1 *ω*9	Nervonic acid	0.35 ± 0.06^a^	0.38 ± 0.07^a^
*ΣMUFA*		**55.97 ± 8.40** ^a^	**55.41 ± 7.20** ^a^
C18:2 *ω*6	Linoleic acid	12.92 ± 1.66^a^	13.07 ± 1.96^a^
C18:3 *ω*3	*α*-Linolenic acid	4.71 ± 0.71^a^	4.70 ± 1.32^a^
C18:3 *ω*6	*γ*-Linolenic acid (GLA)	0.15 ± 0.02^a^	0.32 ± 0.09^b^
C18:4 *ω*3	Stearidonic acid (moroctique)	0.84 ± 0.11^a^	0.82 ± 0.12^a^
C20:4 *ω*6	Eicosatetraenoïc acid (ETA)	0.30 ± 0.04^a^	0.40 ± 0.05^b^
C20:4 *ω*6	Arachidonic acid	0.29 ± 0.06^a^	0.29 ± 0.03^a^
C20:5 *ω*3	Eicosapentaenoïc acid (EPA)	3.05 ± 0.40^a^	3.06 ± 0.46^a^
C22:4 *ω*6	Docosapentaenoïc acid (DPA)	0.13 ± 0.02^a^	0.16 ± 0.03^a^
C22:5 *ω*3	Docosapentaenoïc acid	1.35 ± 0.13^a^	1.30 ± 0.36^a^
C22:5 *ω*6	Docosahexaenoïc acid (DHA)	0.08 ± 0.01^a^	0.10 ± 0.01^a^
C22:6 *ω*3	Docosahexaenoïc acid (DHA)	4.35 ± 0.43^a^	4.30 ± 0.56^a^
*ΣPUFA*		**28.17 ± 3.88** ^a^	**28.52 ± 4.23** ^a^
Total fatty acids		**100.00 ± 0.00** ^a^	**100.0 ± 0.00** ^a^
Number of fatty acids		**26**	**26**
*Σω*3		**14.3 ± 1.86** ^a^	**14.18 ± 2.55** ^a^
*Σω*6		**13.87 ± 1.37** ^a^	**14.34 ± 2.15** ^a^
*Σω*3/*Σω*6		**1.03 ± 0.13** ^a^	**0.98 ± 0.17** ^a^
*Σ*PUFAs/*Σ*SFA		**1.77 ± 0.27** ^a^	**1.78 ± 0.32** ^a^
DHA/EPA		**1.45 ± 0.14** ^a^	**1.44 ± 0.26** ^a^

PUFA: polyunsaturated fatty acids; MUFA: monounsaturated fatty acids; SFA: saturated fatty acids.

In the same row, the numbers with the same letters are not significantly different (*P* > 0.05), *n* =3.

**Table 3:** Antibacterial activity of *Fontitrygon margarita* liver oils according to the extraction method (mg/ml).

Bacteria	Strains code	ATB	Oil extracted by exudation						Oil extracted by cooking-pressure					
				Oil		Nanoemulsion			Oil			Nanoemulsion		
		MIC	MIC	MBC	R	MIC	MBC	r	MIC	MBC	R	MIC	MBC	r
Escherichia coli	ATCC 8739 ATCC10536	16 8	64 32	256 128	4 4	6.25 6.25	50 -	10.24 5.12	64 16	256 64	4 4	6.25 6.25	50 -	10.24 2.56
	EC6	8	16	64	4	6.25	50	2.56	64	128	2	6.25	—	10.24
	EC137	8	16	128	8	6.25	50	2.56	32	256	8	25	—	1.28
Enterobacter cloacae	ENT119	8	16	64	4	6.25	—	2.56	16	64	4	6.25	—	2.56
	ENT 163 ENT51	8 8	64 16	256 128	4 8	6.25 6.25	25 -	10.24 2.56	64 64	128 256	2 4	6.25 6.25	- -	10.24 10.24
Pseudomonas aeruginosa	ATCC27853 PSNEA	8 8	32 32	64 64	2 2	25 6.25	- -	1.28 5.12	32 128	128 256	4 2	12.5 12.5	—	2.61 8
Klebsiella pneumoniae	KLPB 101	4	32	256	8	25	—	1.28	128	256	2	12.5	—	10.24
	KL 11	4	16	128	8	6.25	—	2.56	64	128	2	6.25	—	10.24
Salmonella enterica serovar typhi	ATCC 28579 SAL 9	4 4	16 256	128 -	8 -	6.25 25	- -	2.56 10.24	64 64	256 256	4 4	12.5 6.25	—	5.12 10.24
Citrobacter freundi	CITB 80 CITB 81	16 16	16 64	128 128	8 2	6.25 6.25	-50	2.56 10.24	64 32	256 256	4 8	6.25 6.25	- -	10.24 5.12
Yersinia enterocolitica	YERB 121 YERB 1	16 2	32 16	128 256	2 16	6.25 6.25	- -	5.12 2.56	64 16	256 128	4 8	6.25 6.25	50 -	10.24 2.56
Serratia marcescens	SERB 115	4	16	64	4	6.25	—	2.56	32	128	2	6.25	—	5.12
Staphylococcus aureus	ST 120 MRSA 3	8 32	32 128	256 256	8 2	6.25 6.25	50 -	5.12 20.48	128 128	256 256	2 2	12.5 12.5	25 -	10.24 10.24

ATB: antibiotic, MIC: minimum inhibitory concentration, MBC: minimum bactericidal concentration, R: MBC oil/MIC oil r: MIC Oil/MIC nanoemulsion.

**Table 4:** *Fontitrygon margarita* liver oils inhibition parameters in the presence of MIC and 2 MIC antibiotics on bacteria (mg/ml).

Bacteria	Strains code	MIC ATB	Oil extracted by exudation		Oil extracted by cooking-pressure	
			MIC	2MIC	MIC	2MIC
*CIPROFLOXACIN*						
Escherichia coli	ATCC 8739	8	16(2) ^In^	32(4) ^In^	32(4) ^In^	32(8) ^in^
Enterobacter cloacae	ENTB 51	8	16(2) ^In^	16(2) ^In^	32(4) ^In^	32(8) ^in^
Klebsiella pneumoniae	KL 11	16	32(2) ^In^	16(1) ^Ad^	16(1) ^Ad^	32(2) ^in^
Salmonella typhi	SA L 9	32	32(1) ^Ad^	16(0.5) ^S^	16(0.5) ^S^	8(0.25) ^S^
Citrobacter freundii	CITB 81	16	32(2) ^In^	32(2)^In^	64(4) ^In^	64(2) ^in^
Yersinia enterocolitica	YERB 1	16	4(0.25) ^S^	8(0.5) ^S^	64(4) ^In^	32(2) ^in^
Staphylococcus aureus	ST120	32	32(1) ^Ad^	64(2) ^In^	16(0.5) ^S^	16(0.5) ^S^
*TETRACYCLINE*						
Escherichia coli	ATCC 8739	32	32(1) ^Ad^	16(0.5) ^S^	16(0.5) ^S^	16(0.5) ^S^
Enterobacter cloacae	ENTB 51	16	32(2) ^In^	32(2) ^In^	32(2) ^In^	64(4) ^In^
Klebsiella pneumoniae	KL 11	32	32(1) ^Ad^	32(1) ^Ad^	32(1) ^Ad^	32(1) ^Ad^
Salmonella typhi	SA L 9	32	32(1) ^Ad^	32(1) ^Ad^	16(0.5) ^S^	4(0.125) ^S^
Citrobacter freundii	CITB 81	32	4(0.125) ^S^	4(0.125) ^S^	16(0.5) ^S^	16(0.5) ^S^
Yersinia enterocolitica	YERB 1	2	16(8) ^An^	16(8) ^An^	32(2) ^In^	32(2) ^In^
Staphylococcus aureus	ST120	16	64(4) ^In^	64(4) ^In^	32(2) ^In^	32(2) ^In^
*CHLORAMPHENICOL*						
Escherichia coli	ATCC 8739	32	16(0.5) ^S^	16(0.5) ^S^	64(2) ^In^	64(2) ^In^
Enterobacter cloacae	ENTB 51	16	32(2) ^In^	64(4) ^In^	32(2) ^In^	16(1) ^Ad^
Klebsiella pneumoniae	KL 11	32	8(0.25) ^S^	8(0.25) ^S^	8(0.25) ^S^	8(0.25) ^S^
Salmonella typhi	SA L 9	16	32(2) ^In^	32(2) ^In^	64(4) ^In^	32(2) ^In^
Citrobacter freundii	CITB 81	32	32(1) ^Ad^	16(2) ^In^	16(0.5) ^S^	4(0.25) ^S^
Yersinia enterocolitica	YERB 1	32	16(0.5) ^S^	8(0.25) ^S^	32(1) ^Ad^	4(0.25) ^S^
Staphylococcus aureus	ST120	32	64(2)^In^	64(2)^In^	16(0.5) ^S^	16(0.5) ^S^
*AMOXICILLIN*						
Escherichia coli	ATCC 8739	16	32(2) ^In^	32(2) ^In^	64(4) ^In^	32(2) ^In^
Enterobacter cloacae	ENTB 51	16	32(2) ^In^	16(1) ^Ad^	32(2) ^In^	4(0.25) ^S^
Klebsiella pneumoniae	KL 11	16	64(4) ^In^	64 (4) ^In^	64(4) ^In^	32(2) ^In^
Salmonella typhi	SA L 9	8	16(2) ^In^	16(2) ^In^	64(8) ^An^	32(4) ^In^
Citrobacter freundii	CITB 81	8	16(2) ^In^	16(2) ^In^	16(2) ^In^	32(4) ^In^
Yersinia enterocolitica	YERB 1	8	32(4) ^In^	2(0.25) ^S^	8(1) ^Ad^	4(0.5) ^S^
Staphylococcus aureus	ST120	4	32(8) ^An^	16(4) ^In^	32(8) ^An^	16(4) ^In^
*GENTAMICIN*						
Escherichia coli	ATCC 8739	16	32(2) ^In^	32(2) ^In^	64(4) ^In^	64(4) ^In^
Enterobacter cloacae	ENTB 51	16	32(2) ^In^	32(2) ^In^	32(2) ^In^	32(2) ^In^
Klebsiella pneumoniae	KL 11	8	32(4) ^In^	4(0.5) ^S^	8(1) ^Ad^	8(1) ^Ad^
Salmonella typhi	SA L 9	8	64(8) ^An^	64(8) ^An^	32(4) ^In^	32(4) ^In^
Citrobacter freundii	CITB 81	16	32(2) ^In^	32(2) ^In^	8(0.5) ^S^	8(0.5) ^S^
Yersinia enterocolitica	YERB 1	8	32(4) ^In^	32(4) ^In^	64(8) ^An^	32(4) ^In^
Staphylococcus aureus	ST120	16	16(1) ^Ad^	16(1) ^Ad^	32(2) ^In^	32(2) ^In^

ATB: antibiotic, MIC: minimum inhibitory concentration, An: antagonism, S: synergy, In: indifference, Ad: additive.

## Data Availability

The data used to support the findings of this study are available from the corresponding author upon request.
